# A multi-faceted analysis of disability and poverty in the Polokwane Local Municipality: A narrative review

**DOI:** 10.4102/ajod.v15i0.1744

**Published:** 2026-03-13

**Authors:** Kutu S. Ramolobe

**Affiliations:** 1 Department of Public Management and Leadership, Faculty of Humanities, Nelson Mandela University, Gqeberha, South Africa; 2 Department of Public Management and Governance, Faculty of Management and Public Administration Sciences (FMPAS), Walter Sisulu University, Butterworth, South Africa

**Keywords:** poverty, disability, quality of life, social inclusion, Polokwane local municipality

## Abstract

**Background:**

The interplay between poverty and disability creates a cyclical relationship where disability can increase poverty, and poverty can increase the risk of disability. While extensive research has explored this nexus globally, less is known about its specific impact on the quality of life of persons with disabilities within localised contexts like the Polokwane Local Municipality in South Africa.

**Objectives:**

The purpose of this study is to conduct a multi-faceted analysis of the relationship between disability and poverty in the Polokwane Local Municipality.

**Method:**

This study is a narrative review that utilises a critical synthesis approach to examine the relationship between disability and poverty.

**Results:**

This review reveals that person with disabilities in Polokwane face compounded vulnerabilities, with poverty manifesting as absolute lack of necessities, relative deprivation, situational crises and limited access in rural areas.

**Conclusion:**

This critical review underscores the multi-faceted ways in which disability intersects with various forms of poverty within the Polokwane Local Municipality, highlighting the compounded vulnerabilities faced by persons with disabilities.

**Contribution:**

This article offers a significant contribution to the understanding of the intricate relationship between disability and poverty, specifically within the Polokwane Local Municipality.

## Introduction

The complex interplay between poverty and disability has been extensively documented, with numerous studies highlighting the cyclical nature of their relationship. As the World Health Organization (WHO 2011) notes, disability can increase the risk of poverty, and poverty, in turn, can increase the risk of disability. This study focuses on the Polokwane Local Municipality in South Africa. Almost 6% of the municipal population in Polokwane live with a disability. According to the Community Survey 2007 (Polokwane Local Municipality 2013), there are 33 701 persons with disabilities in Polokwane Local Municipality. The most common forms of disability in Polokwane Local Municipality are physical (18%) and sight (9%).

Existing research has extensively documented how disability can increase the risk of poverty through barriers like limited access to education and employment (Bakhshi, Babulal & Trani 2020; Bredgaarda & Salado-Rasmussen 2021; Graham 2020; Hyde & Livermore 2016; Lang & Upah 2008), and conversely, how poverty can exacerbate the likelihood of disability through factors such as poor health and limited access to essential resources (Banks, Kuper & Polack 2017; Mitra, Posarac & Vick 2013; Nielsen & Midtsundstad 2020). A substantial body of literature has explored this intricate relationship (Bakhshi et al., 2020; Banks et al., 2017; Mitra et al., 2013). Banks et al. (2020) found that persons with disabilities and their families experience poverty and deprivation at higher rates than those without disabilities, a vulnerability particularly pronounced among individuals with cognitive and behavioural impairments, children and working-age adults. Pinilla-Roncancio et al. (2020) further underscored the disproportionate impact of poverty on persons with disabilities, emphasising the need for targeted interventions.

Studies focusing on specific contexts further illuminate this complex relationship. Bakhshi et al. (2020), for instance, revealed a strong correlation between poverty, disability and limited educational access in post-civil war Sierra Leone, perpetuating a cycle of poverty and unemployment. Banks et al. (2017) highlighted the intricate connection between health, disability and poverty, particularly in low- and middle-income countries, where this bidirectional relationship can create a vicious cycle, hindering social and economic advancement for persons with disabilities. Research by Trani et al. (2015) in Morocco and Tunisia demonstrated that persons with disabilities experience higher rates of multidimensional poverty, with unemployment as a significant contributing factor, a situation particularly concerning for women with disabilities who face additional barriers. Mitra et al. (2013) emphasised the diverse pathways through which poverty can increase the risk of disability, stressing the importance of a comprehensive understanding of this multi-faceted relationship.

However, while the link between poverty and disability is clear, the lived experiences and the impact of this intersection on their overall well-being and life satisfaction remain less explored in specific local contexts like the Polokwane local municipality. Understanding how the combined experience of poverty and disability diminishes opportunities for social participation, personal fulfilment and overall well-being is crucial for developing truly inclusive and supportive policies in this region. Quality of life, which has increasingly been recognised as a vital aspect of individual growth and societal aspiration (Hernandez & Bravo 2019), offers a holistic lens through which to understand these impacts. In the health and social sciences, there is a growing emphasis on interventions that enhance individuals’ quality of life (Avolio et al., 2013). Defined broadly in this study as the general circumstances of persons with disabilities, a positive quality of life encompasses a general sense of well-being, a positive sense of social participation and opportunities to achieve personal potential (Schalock et al., 2002 cited in Ramolobe 2023). Therefore, the aim of this narrative review is to conduct a multi-faceted analysis of the relationship between disability and poverty in the Polokwane Local Municipality.

## Methods

In this narrative literature review, we adopted an interpretive and discursive synthesis approach, prioritising depth of understanding and a critical perspective over the narrow, technical focus of a systematic review. The methodology of this study, which aligns with best practices for narrative reviews (Byrne 2016; Greenhalgh, Thorne & Malterud 2018), was designed to confront the complex interplay of disability and poverty within the specific context of the Polokwane Local Municipality.

### Search strategy

The review’s scope was defined by a central research question that guided study’s purposive and judicious selection of evidence. The search strategy for this study was cross-disciplinary, with a particular emphasis on disability studies. The study drew on prior knowledge of the field and conducted searches across multiple academic databases, including Academic Search Complete and JSTOR Arts and Sciences, as well as institutional library resources. The study also included a targeted search for grey literature on the websites of relevant think-tanks and cultural actors to identify barriers and facilitators related to our topic.

The keywords used in the search included terms related to disability, poverty and the geographical location of our study. The study focused on literature published in academic journals and unpublished Doctor of Philosophy (PhD) dissertations. While the study largely focused on sources in English, the study also cited some reports in other languages, acknowledging that language is an inherent limitation of this review. The study did not formally assess the methodological quality of the sources but did exclude blogs and other non-academic online contributions to maintain a scholarly focus.

### Selection criteria

The selection of sources was not guided by a formal set of inclusion or exclusion criteria. Instead, it was an iterative process aimed at identifying relevant and informative materials. The primary consideration was the direct or indirect relevance of the source to the core themes of the review: disability, poverty and their intersection. The article particularly looked for literature that provided distinct frameworks or conceptualisations of poverty, specifically absolute, relative, situational and rural poverty and examined how these frameworks relate to the experiences of persons with disabilities. The inclusion of sources was based on their ability to contribute to a nuanced understanding of these complex dynamics.

### Data analysis and synthesis

**FIGURE 1:**
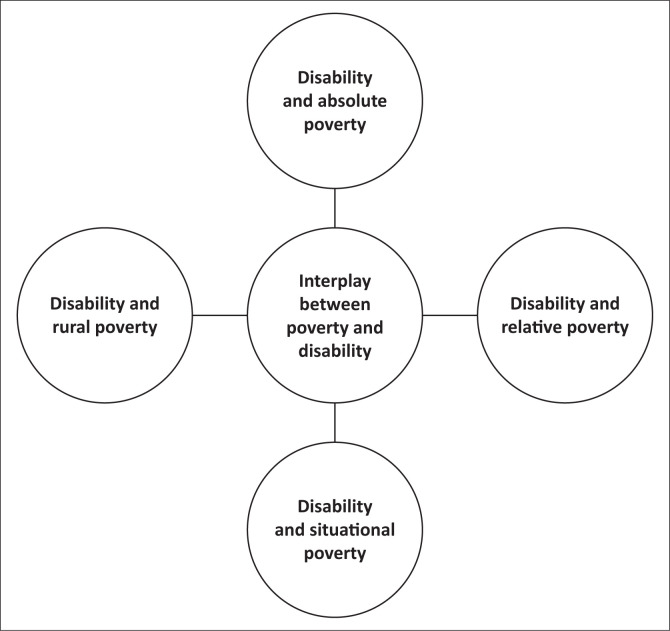
Different facets of poverty.

To address the observation that ‘poverty’ is often treated as a monolithic entity in the literature on disability, we developed a synthesised framework based on different categories of poverty. This framework is not a pre-existing model but was created by synthesising common themes and distinctions from existing literature on poverty studies (e.g. Kim, Lee & Kim 2023; Dillahunt & Veinot 2018; Wan, Hu & Liu 2021). The aim was to provide a more granular understanding of how different facets of poverty (absolute, relative, situational and rural) uniquely impact the quality of life of persons with disabilities in the Polokwane Local Municipality. This multi-faceted approach allows for a deeper analysis, revealing nuances often overlooked in conventional studies and paving the way for more targeted and effective interventions (See Figure 1). In the subsequent sections, we will discuss each of these facets of poverty in detail, using this framework to structure the study’s findings and analysis.

### Ethical considerations

This article followed all ethical standards for research without direct contact with human or animal subjects.

## Review findings

This article confronts the complex interplay of disability and poverty in the Polokwane Local Municipality, revealing nuances often overlooked in conventional analyses. The article confronts the complex interplay of disability and poverty by employing a synthesised framework based on different categories of poverty. This framework, drawing upon common themes and distinctions across various poverty frameworks in existing literature on poverty studies (e.g. Dillahunt & Veinot 2018; Kim, Lee & Kim 2023; Ramolobe 2023; Wan, Hu & Liu 2021; Zhang & Huai 2023), aims to address the observation that ‘poverty’ is often treated as a monolithic entity in the literature on disability and poverty, obscuring the nuanced ways in which different forms of poverty may interact with the specific challenges faced by individuals with disabilities. This framework explores how different facets of poverty, such as lack of basic necessities (absolute poverty), social exclusion (relative poverty), vulnerability to shocks (situational poverty) and geographical disadvantages (rural poverty), uniquely impact persons with disabilities’ quality of life (See Figure 1). By examining these distinct facets, this study aims to provide a more granular understanding that can inform more targeted and effective interventions. In the following sections, the study will discuss the different facets of poverty in more detail.

### Disability and absolute poverty

Persons living in absolute poverty are particularly vulnerable to preventable diseases like malaria, cholera and waterborne illnesses (WHO 2020). Absolute poverty is marked by extreme lack of basic human needs, including shortage of food, clean water, health, sanitation services, housing, education and knowledge (Adeyeye et al. 2023; Khan et al. 2020). Access to these necessities is influenced not only by income but also by the availability of social services (Gordon et al. 2009). Persons with disabilities face even greater challenges within this context. The findings of this critical review underscore the deeply entrenched challenges faced by persons with disabilities living in absolute poverty within the Polokwane Local Municipality. As highlighted by the WHO (2020), individuals in this situation are highly susceptible to preventable diseases because of a lack of basic necessities (Adeyeye et al., 2023; Khan et al., 2020), a situation exacerbated for persons with disabilities who have greater needs across all basic categories (Palmer 2011). Their reliance on specialised healthcare, adapted living conditions and accessible information formats means that the scarcity of resources disproportionately impacts their well-being and opportunities. Furthermore, households with persons with disabilities are disproportionately affected by absolute poverty (Mitra et al. 2013). This is because the additional costs associated with disability, such as healthcare and assistive devices, are compounded by reduced income-earning potential (Eide & Ingstad 2011). The challenges associated with disability and absolute poverty are also evident in the Polokwane Local Municipality. The findings of a research report by the municipality in 2023 reveal that the absence of affordable and accessible transportation, specialised healthcare facilities and inclusive education and employment services perpetuates a cycle of poverty for households with disabled members (Polokwane Local Municipality 2023).

Ramolobe’s (2023) study confirms this reality, with 65% of respondents in Polokwane agreeing that a lack of access to healthcare is a major barrier to the livelihoods of persons with disabilities in rural areas. This aligns with broader national concerns about healthcare inequities despite constitutional guarantees (Coovadia et al., 2009). To address this, increased investment in mobile healthcare clinics and outreach programmes specifically targeting rural areas with high populations of persons with disabilities is crucial. Furthermore, training healthcare professionals on disability inclusion and providing accessible transportation options to existing facilities can significantly improve access (Jones, Morris & Deruyter 2018; Zhou & Parmanto 2019).

Similarly, the finding that 64% of respondents in Ramolobe’s (2023) study identified a lack of access to education as a significant challenge for the livelihoods of persons with disabilities in rural Polokwane (consistent with Ramolobe et al., 2024) demands urgent attention. To counter this, the municipality should prioritise the retrofitting of mainstream schools to be fully inclusive, providing necessary infrastructure and assistive technologies. Additionally, the establishment of accessible vocational training centres tailored to the skills and interests of persons with disabilities can enhance their employability and economic independence. Addressing these systemic barriers through targeted interventions and policy reforms is essential to fostering a more equitable and inclusive society in the Polokwane Local Municipality.

### Disability and relative poverty

Relative poverty, defined as a sense of deprivation based on one’s position relative to the surrounding community, offers a valuable sociological lens (Eskelinen 2009; Zhang & Huai 2023). However, in contexts like Polokwane where significant absolute poverty exists, this concept must be applied with caution, as focusing solely on relative deprivation can obscure the dire lack of basic necessities (Decerf 2022; Wan, Hu & Liu 2021). Disability and poverty are deeply intertwined, with persons with disabilities often being among the poorest and most vulnerable groups (Groce et al., 2011). Their limited access to human and social services not only restricts their ability to earn income but also diminishes their human capital, which includes their health, education and labour participation (Palmer 2011). For many persons with disabilities in Polokwane, where nearly 40% of the population has no income, their reality extends beyond relative deprivation to a fundamental struggle for survival (Polokwane Local Municipality Research Report 2023). This is especially evident in Limpopo, which reports the lowest income for persons with disabilities in the country (Stats SA 2011).

A study in Limpopo revealed the complexities of economic empowerment for persons with disabilities, highlighting a community gardening project that aimed to generate income and improve food security (Tigere & Moyo 2022). While the project successfully created jobs and enhanced the dignity of participants, it faced significant sustainability challenges because of dependence on able-bodied individuals (Tigere & Moyo 2022). Power imbalances between people with and without disabilities led to the exclusion of the former from key decision-making processes, such as wage payments and financial transparency, despite their ownership of the land. This disempowerment also prevented them from contributing innovative ideas to make the gardens more accessible and technologically advanced (Tigere & Moyo 2022). In the Polokwane Local Municipality, the national trend of urban-based persons with disabilities earning more than their rural counterparts is likely mirrored because of a lack of diverse and accessible employment opportunities in rural areas (Department of Social Development 2016; Stats SA 2011). This confinement to low-paying or informal sector jobs further contributes to income inequality and perpetuates the cycle of poverty.

### Disability and situational poverty

Situational poverty, defined as a transient form of poverty linked to a specific event or crisis (Ahmad & Khan 2019; Jensen 2009), can significantly exacerbate the vulnerabilities faced by persons with disabilities. These triggering incidents, such as natural disasters, resource scarcity, divorce or major health issues, are inherently temporary but can have lasting negative consequences, particularly for individuals already living with disabilities (Jensen 2009). The lack of essential resources like food and clean water following a disaster can worsen pre-existing chronic health conditions (Ningrum, Hukom & Adiwijaya 2020).

The precarious position of persons with disabilities during crises is further underscored by Moore (2013), who argues that their voices are often marginalised because of discrimination, especially in global conflicts and disasters. This vulnerability extends to an increased risk of abuse, particularly in developing countries (UN 2006). Neille and Penn (2015) highlight that this susceptibility to abuse in rural regions stems from historical and contemporary factors, leading to direct harm and indirect social exclusion, deprivation, poverty and degradation. Furthermore, Singal et al. (2015) emphasise that factors closely linked to poverty, such as communicable diseases, childhood illnesses, food shortages and poor sanitation, are also major contributors to disability.

The intersection of situational poverty and disability is evident in various contexts. For instance, the World Bank (2005, 2007) notes a higher prevalence of disability in post-conflict countries and regions prone to natural disasters. The coronavirus disease 2019 (COVID-19) pandemic in South Africa further illustrated this interplay. Mulibana (2020) points out the initial lack of accommodation for essential health and disability services during strict lockdowns, placing disabled individuals at greater risk. McKinney, McKinney and Swartz (2020) highlight the exclusion of crucial services like sign language interpretation and assistive technology in health settings. Huisman (2020) adds that persons with disabilities often face challenges in hospitals because of the unavailability of necessary support services.

The challenges faced in South Africa during the pandemic align with global concerns raised by the WHO and World Bank (2011), which report that 15% of the world’s population lives with a disability, a number growing because of various factors, including disasters. This report also emphasises that disability disproportionately affects vulnerable populations. The Council for Scientific and Industrial Research (CSIR 2023) further highlights the vulnerabilities within the Polokwane local municipality, noting that rapid urbanisation, climate-related pressures like floods and heatwaves and unequal economic growth exacerbate health threats, particularly for vulnerable groups including the poor and the sick. Natural disasters in this context can have immediate and long-term health impacts, including mental health issues, especially when emergency services and essential infrastructure are compromised (CSIR 2023).

### Disability and rural poverty

The intricate relationship between disability and poverty is amplified in rural settings, characterised by populations under 50 000, a higher prevalence of single-parent families, and restricted access to essential facilities, disability care and education (Hiratani & Hohashi 2021; Jensen 2009; Kim, Lee & Kim 2023). Livelihoods in these areas often depend on agriculture and limited local job opportunities, making the transition from welfare to employment challenging because of scarce economic prospects. Consequently, rural poverty rates are escalating, surpassing those in urban areas. Notably, the International Labour Organization (ILO 2007) indicates a higher concentration of persons with disabilities in rural regions, where high poverty, unemployment and limited access to services contribute to underdevelopment and poor living conditions, disproportionately affecting individuals with disabilities (McDaniels, Harley & Beach 2018; Ramolobe 2023).

Experiences from other countries underscore the challenges faced by persons with disabilities in rural environments. In Austria, difficulties with accessibility and access to services are significant, often because of the absence or inconsistency of public transport, necessitating reliance on private transport and assistance from others to reach essential services like healthcare (Dillahunt & Veinot 2018; Pini et al., 2011; Velho 2019). Similarly, Aboriginal Australians in remote areas like West Kimberley experience significant income disparities and severe poverty (Office of Aboriginal Health 2003). Furthermore, the lack of safe infrastructure, such as protected pavements, in Australian rural communities restricts the mobility of persons with disabilities (Pini et al. 2011, cited in Ramolobe 2023). In India, lower employment rates (38.4%) have been observed for persons with disabilities in rural areas (Mitra & Sambamoorthi 2013). Moreover, the inadequate quality of education in rural schools in countries like Malawi and Botswana has a greater impact on disabled youth, compounded by transportation barriers to access specialised schooling (Booysens, Van Pletzen & Lorenzo 2015).

In the context of South Africa, persons with disabilities in rural areas face marginalisation and exclusion from most developmental initiatives, encountering significant economic, political and social obstacles (Tigere & Moyo 2022). Specifically, within the Polokwane Local Municipality, accessing livelihoods is a major challenge for persons with disabilities because of a lack of transportation, limited job opportunities in rural areas and insufficient participation (Ramolobe 2023). The often limited or non-existent public transport in these rural areas directly hinders the ability of persons with disabilities to reach employment and other essential services (Ramolobe 2023). Furthermore, research by Ramolobe (2023) indicates a lack of support services for persons with disabilities in the rural parts of the Polokwane Local Municipality. The transportation infrastructure in the municipality further exacerbates these issues, with approximately 800 km of gravel roads compared to 567 km of paved roads, posing significant challenges for accessibility, particularly for individuals with mobility impairments. The municipality also grapples with storm water management, which can further impede movement and threaten infrastructure (Polokwane Local Municipality 2019).

## Conclusion and recommendations

In conclusion, this critical review has illuminated the multi-faceted interplay between disability and poverty, moving beyond a monolithic understanding of poverty to explore its distinct dimensions (absolute, relative, situational and rural poverty) and their unique impacts on persons with disabilities. The analysis reveals that persons with disabilities in Polokwane face compounded vulnerabilities. Those in absolute poverty experience a greater lack of basic necessities, exacerbating health risks and limiting access to essential services. While relative poverty offers a sociological lens on deprivation, its significance must be weighed against the stark realities of absolute poverty prevalent in the municipality. Situational poverty, arising from crises, disproportionately affects persons with disabilities because of their marginalised status and potential lack of accessible support during emergencies. Finally, rural poverty creates a particularly challenging environment because of limited access to infrastructure, employment opportunities and support services, further isolating and impoverishing individuals with disabilities. Ultimately, this review underscores the urgent need for targeted and nuanced interventions that address the specific ways in which different forms of poverty intersect with disability in the Polokwane Local Municipality to foster a more inclusive and equitable society. This review, while providing a synthesised understanding of the interplay between disability and poverty in the Polokwane Local Municipality, is subject to certain limitations. The reliance on existing literature, including reports and studies conducted within South Africa and internationally, means that the specific nuances and localised data within Polokwane may not be exhaustively covered. The limited availability of disaggregated data on disability across different poverty categories within the municipality could also restrict the depth of the analysis. Furthermore, the review primarily focuses on the four identified categories of poverty and may not fully capture the complexity of poverty as a multidimensional phenomenon. Future research that directly engages with individuals with disabilities and collects primary data within the Polokwane context will be essential to overcome these limitations and provide a more comprehensive and nuanced understanding of this critical issue.

## References

[CIT0001] Adeyeye, S.A.O., Ashaolu, T.J., Bolaji, O.T., Abegunde, T.A. & Omoyajowo, A.O., 2023, ‘Africa and the Nexus of poverty, malnutrition and diseases’, *Critical Reviews in Food Science and Nutrition* 63(5), 641–656. 34259104 10.1080/10408398.2021.1952160

[CIT0002] Ahmad, M. & Khan, A.U., 2019, ‘Global economic impact of antibiotic resistance: A review’, *Journal of Global Antimicrobial Resistance* 19, 313–316. 31176071 10.1016/j.jgar.2019.05.024

[CIT0003] Bakhshi, P., Babulal, G. & Trani, J.F., 2020, ‘Disability, poverty, and schooling in post-civil war in Sierra Leone’, *European Journal of Development Research* 33(10), 482–501.

[CIT0004] Banks, L.M., Hameed, S., Usman, S.K. & Kuper, H., 2020, ‘No one left behind? Comparing poverty and deprivation between people with and without disabilities in the Maldives’, *Sustainability* 12(5), 2066.

[CIT0005] Banks, L.M., Kuper, H. & Polack, S., 2017, ‘Poverty and disability in low- and middle-income countries: A systematic review’, *PLoS One* 12(12), e0189996. 10.1371/journal.pone.0189996 29267388 PMC5739437

[CIT0006] Booysens, M., Van Pletzen, E. & Lorenzo, T., 2015, ‘The complexity of rural contexts experienced by community disability workers in three southern African countries’, *African Journal of Disability* 4(1), 167. 28730029 10.4102/ajod.v4i1.167 PMC5433477

[CIT0007] Braithwaite, J. & Mont, D., 2008, ‘Disability and poverty: A survey of World Bank poverty assessments and implications’, *Alter* 3, 219–232.

[CIT0008] Bredgaard, T. & Salado-Rasmussen, J., 2021, ‘Attitudes and behaviour of employers to recruiting persons with disabilities’, *Alter* 15(1), 61–70. 10.1016/j.alter.2020,04.004

[CIT0009] Byrne, J.A., 2016, ‘Improving the peer review of narrative literature reviews’, *Research Integrity and Peer Review* 1(1), 12. 29451529 10.1186/s41073-016-0019-2 PMC5803579

[CIT0010] Coovadia, H., Jewkes, R., Barron, P., Sanders, D. & McIntyre, D., 2009, ‘The health and health system of South Africa: Historical roots of current public health challenges’, *The Lancet* 374(9692), 817–834. 10.1016/S0140-6736(09)60951-X 19709728

[CIT0011] Council for Scientific and Industrial Research (CSIR), 2023, *Polokwane Local Municipality: Climate risk profile report*, GIZ, DFFE, DHS, the HDA & City of Polokwane, viewed 20 April 2025, from https://greenbook.co.za/documents/GIZ_RiskProfile_PolokwaneLM_Sep2023.pdf.

[CIT0012] Decerf, B., 2022, ‘Absolute and relative poverty measurement’, *Development Research*, Policy research working paper 1008, viewed 29 October 2025, from https://pdfs.semanticscholar.org/35a8/3a71ee9f637ab2c4cc567f22f34096d85c1e.pdf.

[CIT0013] Dillahunt, T.R. & Veinot, T.C., 2018, ‘Getting there: Barriers and facilitators to transportation access in underserved communities’, *ACM Transactions on Computer-Human Interaction (TOCHI)* 25(5), 1–39.

[CIT0014] Dubois, J.L. & Trani, J.F., 2009, ‘Extending the capability paradigm to address the complexity of disability’, *Alter* 3(3), 192–218.

[CIT0015] Eskelinen, T., 2009, *Putting global poverty in context: A philosophical essay on power, justice and economy*, University of Jyvaskyla, Jyvaskyla.

[CIT0016] Gordon, D., Irving, M.K., Nandy, S. & Townsend, P., 2009, *The extent and nature of absolute poverty. Final report to DFID: R8382*, The University of Bristol, s. 4, viewed 25 August 2022, from https://assets.publishing.service.gov.uk/media/57a08c37ed915d622c0011d7/R8382F.pdf.

[CIT0017] Graham, L., Moodley, J., Ismail, Z., Munsaka, E., Ross, E. & Schneider, M., 2014, *Poverty and disability in South Africa*, Centre for Social Development in Africa, University of Johannesburg, Johannesburg.

[CIT0018] Greenhalgh, T., Thorne, S. & Malterud, K., 2018, ‘Time to challenge the spurious hierarchy of systematic over narrative reviews?’, *European Journal of Clinical Investigation* 48(6), e12931. 29578574 10.1111/eci.12931 PMC6001568

[CIT0019] Hiratani, Y. & Hohashi, N., 2021, ‘Family functioning of single-parent families with children attending a special needs school in Japan’, *Pediatrics International* 63(5), 581–588. 32996283 10.1111/ped.14486

[CIT0020] Huisman, B., 2020, *COVID-19: Life under lockdown for people living with disabilities*, Spotlight, viewed 10 April 2025, from https://www.spotlightnsp.co.za/2020/05/19/covid-19-life-under-lockdownfor-people-living-with-disabilities/.

[CIT0021] Jensen, E., 2009, *How poverty affects behavior and academic performance. Teaching with poverty in mind*, viewed 20 August 2025, from https://www.csun.edu/~sb4310/Clsrmangment/Jensench2.pdf.

[CIT0022] Jones, M., Morris, J. & Deruyter, F., 2018, ‘Mobile healthcare and people with disabilities: Current state and future needs’, *International journal of environmental research and Public Health* 15(3), 515. 29538292 10.3390/ijerph15030515 PMC5877060

[CIT0023] Khan, N., Naushad, M., Faisal, S. & Fahad, S., 2020, *Analysis of poverty of different countries of the world*, viewed 29 April 12025, from https://papers.ssrn.com/sol3/papers.cfm?abstract_id=3701329.

[CIT0024] Kim, H.S., Lee, C.E. & Kim, K.M., 2023, ‘Challenges of single parents raising children with intellectual and developmental disabilities’, *Journal of Applied Research in Intellectual Disabilities* 36(4), 777–786. 36896654 10.1111/jar.13093

[CIT0025] McDaniels, B.W., Harley, D.A. & Beach, D.T., 2017, ‘Transportation, accessibility, and accommodation in rural communities’, in *Disability and vocational rehabilitation in rural settings: Challenges to service delivery*, pp. 43–57, Springer International Publishing, Cham.

[CIT0026] McKinney, E.L., McKinney, V. & Swartz, L., 2021, ‘Access to healthcare for persons with disabilities in South Africa: Bad at any time, worse during COVID-19?’, *South African Family Practice* 63(1), a5226. 10.4102/safp.v63i1.5226 PMC8335793 34342484

[CIT0027] Mitra, S., Posarac, A. & Vick, B., 2013, ‘Disability and poverty in developing countries: A multidimensional study’, *World Development* 41(C), 1–18.

[CIT0028] Mitra, S. & Sambamoorthi, U., 2013, ‘Disability prevalence among adults: estimates for 54 countries and progress toward a global estimate’, *Disability and Rehabilitation* 36(11), 940–947 23962193 10.3109/09638288.2013.825333

[CIT0029] Moore, M., 2013, ‘Disability, global conflicts and crises’, *Disability and Society* 28(6), 741–743.

[CIT0030] Mulibana, M., 2020, *Lack of consultation led to persons with disabilities being neglected in the COVID-19 response*, AfricLaw, viewed 10 April 2025, from https://africlaw.com/2020/05/18/lack-of-consultation-led-to-persons-with-disabilities-being-neglected-in-the-covid-19-response/.

[CIT0031] Neille, J. & Penn, C., 2015, ‘Beyond physical access: A narrative analysis into the barriers to policy implementation and service provision experienced by people living in a rural context’, *Rural and Remote Health* 15, 3332. 26268958

[CIT0032] Nielsen, L., 2009, *Global relative poverty*, IMF Working Paper WP/09/03, International Monetary Fund, New York, NY.

[CIT0033] Nielsen, R.A. & Midtsundstad, T.I., 2020, ‘Do workplace health-promotion interventions targeting employees with poor health reduce sick-leave probability and disability rates?’, *Scandinavian Journal of Public Health* 49(2), 219–227. 32807034 10.1177/1403494820946543 PMC7917567

[CIT0034] Ningrum, P.A., Hukom, A. & Adiwijaya, S., 2020, ‘The Potential of Poverty in the City of Palangka Raya: Study SMIs Affected Pandemic Covid 19’, *Budapest International Research and Critics Institute-Journal (BIRCI-Journal)* 3, 1626–1634.

[CIT0035] Palmer, M., 2011, ‘Disability and poverty: A conceptual review’, *Journal of Disability Policy Studies* 21(4), 210–218.

[CIT0036] Pini, B., Soldatic, K., Meekosha, H. & Thomas, C., 2011, *Disability in rural Australia*, DP 110102710, Australian Research Council, Melbourne.

[CIT0037] Pinilla-Roncancio, M., 2015, ‘Disability and poverty: Two related conditions. A review of the literature’, *Revista de la Facultad de Ciencias Medicas de Cordoba* 63, 113–123. 10.15446/revfacmed.v63n3sup.50132

[CIT0038] Pinilla-Roncancio, M., Mactaggart, I., Kuper, H., Dionicio, C., Naber, J., Murthy, G.V.S. et al., 2020, ‘Multidimensional poverty and disability: A case control study in India, Cameroon, and Guatemala’, *SSM-Population Health* 11, 100591. 10.1016/j.ssmph.2020.100591 32405529 PMC7212179

[CIT0039] Polokwane Local Municipality, 2023, *2030 economic growth and development plan: Research report*, viewed 20 April 2025, from https://www.polokwane.gov.za/wp-content/uploads/2023/07/Research-Report.pdf.

[CIT0040] Ramolobe, K.S., 2023, ‘The effects of poverty on the quality of life of people with disabilities: A case of Polokwane local municipality’, Unpublished doctoral thesis, Nelson Mandela University.

[CIT0041] Tigere, B. & Moyo, T., 2022, ‘Disability-inclusive community development: A case of a community garden in Limpopo province in South Africa’, *African Journal of Disability* 11, a850. 10.4102/ajod.v11i0.850 PMC8831988 35169548

[CIT0042] Trani, J.F., Bakhshi, P., Tlapek, S.M., Lopez, D. & Gall, F., 2015, ‘Disability and poverty in Morocco and Tunisia: A multidimensional approach’, *Journal of Human Development and Capabilities* 16(4), 518–548.

[CIT0043] Trein, P. & Wagner, J., 2021, ‘Governing personalized health: A scoping review’, *Frontiers in Genetics* 12, 650504. 33968134 10.3389/fgene.2021.650504 PMC8097042

[CIT0044] Velho, R., 2019, ‘Transport accessibility for wheelchair users: A qualitative analysis of inclusion and health’, *International Journal of Transportation Science and Technology* 8(2), 103–115.

[CIT0045] Wan, G., Hu, X. & Liu, W., 2021, ‘China’s poverty reduction miracle and relative poverty: Focusing on the roles of growth and inequality’, *China Economic Review* 68, 101643.

[CIT0046] World Bank, 2007, *First ever regional catastrophe risk insurance pool up and running in time for 2007 hurricane season*, World Bank, New York, NY.

[CIT0047] World Health Organization, 2001, *International classification of functioning, disability and health*, WHO, Geneva.

[CIT0048] World Health Organization, 2011, *World disability report and rehabilitation*, WHO, Geneva.

[CIT0049] World Health Organization, 2020, *Children: Improving survival and wellbeing*, WHO, Geneva.

[CIT0050] World Health Organization & World Bank, 2011, *World report on disability*, WHO, Geneva, viewed 25 August 2022, from http://www.who.int/disabilities/world_report/2011/report.pdf.

[CIT0051] Zhang, Y. & Huai, J., 2023, ‘A case study of farmers’ behavioral motivation mechanisms to crack the fractal multidimensional relative poverty trap in Shaanxi, China’, *Agriculture* 13(11), 2043.

[CIT0052] Zhou, L. & Parmanto, B., 2019, ‘Reaching people with disabilities in underserved areas through digital interventions: Systematic review’, *Journal of Medical Internet Research* 21(10), e12981. 31654569 10.2196/12981 PMC7380899

